# PdpC, a secreted effector protein of the type six secretion system, is required for erythrocyte invasion by *Francisella tularensis* LVS

**DOI:** 10.3389/fcimb.2022.979693

**Published:** 2022-09-27

**Authors:** Stuart Cantlay, Christian Kaftanic, Joseph Horzempa

**Affiliations:** Department of Biological Sciences, West Liberty University, West Liberty, WV, United States

**Keywords:** *Francisella tularensis*, T6SS, effector, PdpC, erythrocyte invasion

## Abstract

*Francisella tularensis* is a gram negative, intracellular pathogen that is the causative agent of the potentially fatal disease, tularemia. During infection, *F. tularensis* is engulfed by and replicates within host macrophages. Additionally, this bacterium has also been shown to invade human erythrocytes and, in both cases, the Type Six Secretion System (T6SS) is required for these host-pathogen interaction. One T6SS effector protein, PdpC, is important for macrophage infection, playing a role in phagolysosomal escape and intracellular replication. To determine if PdpC also plays a role in erythrocyte invasion, we constructed a *pdpC*-null mutant in the live vaccine strain, *F. tularensis* LVS. We show that PdpC is required for invasion of human and sheep erythrocytes during *in vitro* assays and that reintroduction of a copy of *pdpC*, *in trans*, rescues this phenotype. The interaction with human erythrocytes was further characterized using double-immunofluorescence microscopy to show that PdpC is required for attachment of *F. tularensis* LVS to erythrocytes as well as invasion. To learn more about the role of PdpC in erythrocyte invasion we generated a strain of *F. tularensis* LVS expressing *pdpC-emgfp*. PdpC-EmGFP localizes as discrete foci in a subset of *F. tularensis* LVS cells grown in broth culture and accumulates in erythrocytes during invasion assays. Our results are the first example of a secreted effector protein of the T6SS shown to be involved in erythrocyte invasion and indicate that PdpC is secreted into erythrocytes during invasion.

## Introduction

*Francisella tularensis* is gram-negative, pathogenic bacterium that is of concern to human health as the agent of the zoonotic disease, tularemia (Ellis et al., 2002; Keim et al., 2007). It can be highly infectious, with as few as 10 bacteria being able to initiate a potentially lethal infection (Conlan and Oyston, 2007) and as a result has been designated as a category A biodefense agent (Dennis et al., 2001). The primary route of pathogenesis for *F*. *tularensis* is entry into host macrophages by looping phagocytosis (Clemens et al., 2005) followed by phagolysosomal escape and intracellular proliferation (Anthony et al., 1991; Fortier et al., 1995; Golovliov et al., 2003; Clemens et al., 2004). *F. tularensis* can infect a variety of other host cell types through endocytic pathways, including lung alveolar cells, kidney cells, hepatocytes and fibroblasts (Fujita et al., 1993; Bosio and Dow, 2005; Qin and Mann, 2006; Hall et al., 2007; Craven et al., 2008; Hall et al., 2008) and *F. tularensis* can be found in blood plasma during infection (Forestal et al., 2007) . *F. tularensis* is one of the few bacterial pathogens that has been shown to be able to invade red blood cells (Horzempa et al., 2011; Schmitt et al., 2017). Whilst *F. tularensis* does not replicate in red blood cells, invasion of host erythrocytes is believed to aid persistence in the host, evade host immune responses and protect bacteria from antibiotics (Horzempa et al., 2011). Also, biting arthropods are key vectors of transmission for *F. tularensis* (Petersen et al., 2009) with ticks being a primary vector in North America (Mörner, 1992) and ticks and mosquitoes acting as vectors in Europe (Mörner, 1992; Thelaus et al., 2014). It is believed that entry into erythrocytes may protect bacteria from the harsh environment of the arthropod gut (Horzempa et al., 2011) and therefore facilitate transmission *via* these vectors. In the wild, *Francisella* spp. have a broad range of hosts including birds and fish (Mörner, 1992), small mammals such as lagomorphs and rodents (Ellis et al., 2002; Schulze et al., 2016) and sheep (O’toole et al., 2008). Transmission of *F. tularensis* to humans can either be through close contact with infected animals or directly transmitted by biting arthropods (Mörner, 1992; Feldman et al., 2001; Sjöstedt, 2007; Gürcan, 2014) so the ability of *F. tularensis* to invade and reside in erythrocytes is of significance for the spread of tularemia in reservoir species and humans.

In contrast to uptake by host phagocytic cells, erythrocytes do not possess any endocytic machinery (Blanton et al., 1968; Schekman and Singer, 1976) and the mechanisms by which *F. tularensis* invades erythrocytes has not yet been fully elucidated. It is known that components of host complement are involved in invasion (Horzempa et al., 2011) and that the type six secretion system (T6SS) is required for efficient invasion of erythrocytes by *F. tularensis* (Schmitt et al., 2017). The T6SS is produced by many gram-negative bacteria (Bingle et al., 2008) and is required for the secretion of effector proteins across the inner and outer membranes into a variety of target cells, including other bacteria or eukaryotic hosts.

The T6SS of *Francisella* is distinct from those found in other bacteria, representing a unique noncanonical group (T6SS^ii^) with many proteins lacking sequence homology to T6SS proteins from other species but possessing similar structural and functional properties (Clemens et al., 2018). The Francisella T6SS is encoded in a gene cluster referred to as the Francisella Pathogenicity Island (FPI) which encodes both structural components of the T6SS and some secreted effector proteins and is present in two copies in the *F. tularensis* genome (Nano and Schmerk, 2007). Expression of the FPI during infection is dependent on recruitment of the σ^70^-associated RNAP holoenzyme to the FPI promoter by the MglA/SspA heterodimer, a third regulatory protein PigR and the stress response signal ppGpp which work in concert to coordinate expression of virulence genes under conditions experienced by *F. tularensis* in host macrophages (Lauriano et al., 2004; Charity et al., 2007; Charity et al., 2009; Wrench et al., 2013; Cuthbert et al., 2015; Cuthbert et al., 2017; Travis et al., 2021). However, the expression of the T6SS in response to encountering erythrocytes is less clear. Although the abundance of iron has been shown to affect the transcription of FPI genes, their expression is seemingly unaffected by the presence of erythrocytes (Deng et al., 2006; Schmitt et al., 2017). Interestingly though, *mglA* and structural components of the T6SS are required for erythrocyte invasion which could suggest that these proteins are regulated post-transcriptionally (Schmitt et al., 2017).

The requirement for the T6SS in pathogenicity of *Francisella* spp. is well established (Lindgren et al., 2004; Nano et al., 2004; de Bruin et al., 2007; Bröms et al., 2010; Robertson et al., 2013; Brodmann et al., 2017) and most recently attention has been focused on the role of secreted effector proteins of the T6SS. Several proteins have been shown to be secreted in an T6SS dependent fashion, some of these are structural components of the T6SS but genetic and functional studies have identified proteins that are likely to be secreted to modulate host cell responses. The FPI proteins, PdpC, PdpD, and IglG in addition to OpiA, OpiB-1 and OpiB-3 which are encoded elsewhere in the genome are all required for full virulence in *Francisella* spp. (Ludu et al., 2008; Lindgren et al., 2013; Long et al., 2013; Eshraghi et al., 2016; Brodmann et al., 2017; Brodmann et al., 2021). IglE, which is predicted to be a membrane attached lipoprotein, was identified as a potential substrate of the T6SS and is also required for the pathogenesis of *F. tularensis* Shcu S4 (Bröms et al., 2012, Robertson et al., 2013). Of all the secreted effector proteins so far characterized, PdpC, in particular, has been shown to be important for virulence and is required for efficient escape from the phagosome, intracellular replication and activation of the AIM2 inflammatory response (Long et al., 2013; Brodmann et al., 2017; Brodmann et al., 2021).

Because the T6SS is required for erythrocyte invasion we are interested in the role of specific effector proteins during this process. We used an attenuated strain, *F*. *tularensis* LVS, that can be handled safely in BLS2 conditions but that retains pathogenicity in some animal models and has two copies of the FPI (Eigelsbach and Downs, 1961; Tigertt, 1962; Elkins et al., 1996). Our results, presented here, show that PdpC is required for efficient erythrocyte invasion for *F. tularensis* LVS and as such is the first secreted effector protein identified to be involved in red blood cell invasion. We also show that a *pdpC-*null strain is affected in its ability to attach to erythrocytes compared to the wild type strain. Using a fluorescence fusion of *pdpC* to *emgfp* we report on the sub-cellular localization of PdpC-EmGFP in *F. tularensis* LVS and show that PdpC-EmGFP fluorescence accumulates in human erythrocytes suggesting that PdpC is secreted directly into red blood cells during invasion.

## Methods

### Bacterial strains, plasmids, and growth conditions

Bacterial strains used in this study are listed in **Table 1**. For cultivation of *F. tularensis* LVS strains, frozen stock cultures of bacteria were streaked onto chocolate II agar (Horzempa et al., 2011) and incubated at 37°C with 5% CO_2_ for 3 to 7 days. For broth cultures, bacteria from plates were used to inoculate either Chamberlain’s chemically defined medium (CDM; pH 6.3) (Chamberlain, 1965), tryptic soy broth (Becton, Dickinson, and Co.) supplemented with 0.1% cysteine hydrochloride (TSBc; Fisher Scientific), or brain heart infusion broth (BHI; Oxoid, Ltd.) supplemented with 0.2% cysteine hydrochloride (pH 6) (Brodmann et al., 2017) and grown to stationary phase by overnight incubation at 37°C with agitation. Where required, media were supplemented with antibiotics at the following concentrations: kanamycin (10 µg ml^-1^), hygromycin (200 µg ml^-1^), or polymyxin B (100 µg ml^-1^). For *E. coli*, bacteria were grown at 37°C on Luria-Bertani (LB) agar plates or in LB broth (Fisher Scientific) with antibiotics where appropriate at the following concentrations: kanamycin (50 µg ml^-1^), hygromycin (200 µg ml^-1^), ampicillin (100 µg ml^-1^), or polymyxin B (100 µg ml^-1^).

**Table 1:** Bacterial strains, plasmids and oligonucleotides used in this study.

Bacterial Strains	Description	Source or reference
***F. tularensis* strains**		
LVS	*Francisella tularensis* subsp. *holarctica* live vaccine strain	The *F. tularensis* LVS strain was a personal gift from Karen Elkins (US Food and Drug Administration)
LVS *pdpC*-null	LVS with one copy of *pdpC* deleted and one copy disrupted	This study
LVS pFNLTP8	LVS containing the *F. tularensis* shuttle vector pFNLTP8	Cantlay et al., 2020
LVS pABST	LVS containing the *F. tularensis* shuttle vector containing the *groEp* promoter, pABST	This study
LVS *pdpC*-null pABST	The *pdpC*-null strain containing pABST	This study
LVS pCTK1	LVS containing the *pdpC* complementation vector pCTK1	This study
LVS *pdpC*-null pCTK1	The *pdpC*-null strain containing the *pdpC* complementation vector pCTK1	This study
LVS pSC13	LVS containing promoterless *emgfp* on pSC13	Cantlay et al., 2020
LVS pSC27	LVS containing *FGRp*-*pdpC-emgfp* on pSC27	This study
LVS pKHEG	LVS expressing *emgfp*	Cantlay et al., 2020
LVS *pdpC*-null pKHEG	The *pdpC*-null strain expressing *emgfp*	This study
LVS pSC25	LVS containing *FGRp-ftsZ-emgfp* on pSC25	This study
LVS Δ*mglA*	LVS with the *mglA* gene deleted	Schmitt et al., 2017
LVS Δ*mglA* pSC27	LVS with the *mglA* gene deleted containing *FGRp*-*pdpC-emgfp* on pSC27	This study
***E. coli* strains**		
DH5α	*fhuA2* Δ(*argF-LacZ*)*U169 phoA glnV44* φ*80*Δ(*lacZ*)*M15 gyrA96 recA1 relA1 endA1 thi-1 hsdR17*	NEB
**Plasmids**		
pJH1	An integrating suicide vector with an I-Sce1 restriction site	Horzempa et al., 2010
pGUTS	A stably replicating plasmid with I-SceI under the control of *FGRp*	Horzempa et al. 2010
pSC1	pJH1 with the center and right 500 bp flanking regions of *pdpC* cloned into the *Bam*HI *Pst*I site of pJH1	This Study
pSC7	pJH1 with an cat *mcherry* cassette flanked by 500 bp of *pdpC* sequence cloned into the *Xma*I *Sph*I site	This study
pFNLTP8	*Francisella* shuttle plasmid	Maier et al., 2004
pABST	*Francisella* shuttle plasmid containing the *groEp* promoter	Robinson et al., 2015
pCTK1	The *pdpC* complementation vector created by cloning a 4,048-bp fragment containg *pdpC* into the *Eco*RI *Sal*I site of pABST	This study
pSC13	*emgfp* cloned into the *Nde*I *Bam*HI site of pFNLTP8	Cantlay et al., 2020
pSC18	*emgfp* cloned downstream of the *FGRp* promoter allowing cloning into the *Eco*RI *Nde*I site to make in frame fusions to *emgfp* driven by *FGRp*.	Cantlay et al., 2020
pSC25	*FGRp pdpC-emgfp* created by cloning the entire coding sequence of *ftsZ into the EcoRI site of pSC18*	This study
pSC27	*FGRp pdpC-emgfp* created by cloning the entire coding sequence of *pdpC* into the *Eco*RI *Nde*I site of pSC18	This study
pKHEG	326 nucleotides, including *FGRp*, from pTC3D cloned into the *Kpn*I *Nde*I site of pSC13	Cantlay et al., 2020
**Oligonucleotides**		
0116F1	CATGGGATCCGAAAATTACCTTTATCATAATTATTA	Integrated DNA Technologies (IDT)
0116R1	TAATAATAAGTCGACGGTACCACCGGTGTTAAGGATACAAATATATGAGTAAA	IDT
0116F2	ACCGGTGGTACCGTCGACTTATTATTAATGTGCCTCCTTAATTTATCAGATAG	IDT
0116R2	CATGCTGCAGCTCTAAATGTAAAAATAAATATC	IDT
SC4	GGTGGT GAATTC GAAAAATCCGAATCAC	IDT
SC5	GGTGGT GTCGAC CATATATTTGTATCCTTAAC	IDT
SC25	CATGCCCGGGGTTCTTTATC	IDT
SC26	CATGGCATGCCCTAAAACAG	IDT
SC31	GGTGGTGAATTCGTTAAGTAGTTTAAAAGGTG	IDT
SC33	GGTGGTGAATTCGCTCCGGGCCCGGCAGCTCCGGGCCCGGCAG TCTTCTTCTTAAGAAACTAG	IDT
SC71	GGTGGTCATATGCTCCGGGCCCGGCAGTGA TGATGATATTTTTTTAAAAAAG	IDT
SC72	GGTGGTGAGCTCTGTAATTGCTGATGATGAC	IDT
SC73	GGTGGTGAGCTCAAGAAGCCAGGAAG	IDT
*pdpC cat mCherry* gBlock®	CATGGGATCCGTTCTTTATCTGGTGAGTATAAGTTGAAATCTTTTTCTATCTGT	IDT
	TTTTTATGAGAAGAGGAGTATTTTGCACCACTATCATACATTATGCCTAGTGA	
	TACAAAACGTTGATATTGATTAATAGCAGCATAGCCATTGTAGTTGTTTACGA	
	CTTTTATTTTCTCTCCATCTTGGACCAATCCTGTCTCCGCTTTCTTCTTACGAGA	
	ATACTGCTCTAATAAAGTACAGTTTCTTTCACTATGAGTTGATAATAGGTTATA	
	CAAGGATCCTTCTGATAGAGAACTATATAAGCAATTTAAATTATATATATCTT	
	CATATACCTCGGATAACGTCGTTACTCTACTTATATAATTTTCTTTGAGAACTT	
	TTGAATTAGGTTTTATGCTATTTTCATACTTTTCAACTAATGCTTTAAACTCTTT	
	ATCCTTAGAAAGTTTAAATACAGGTCTAGCAAAAAAATTAGCTATATTGGCTA	
	TAGGATACTTTTCCTCTTTAGAATTTTTACTTGTACAGCTCGTCCATGCCGCCG	
	GTGGAGTGGCGGCCCTCGGCGCGTTCGTACTGTTCCACGATGGTGTAGTCCTC	
	GTTGTGGGAGGTGATGTCCAACTTGATGTTGACGTTGTAGGCGCCGGGCAGCT	
	GCACGGGCTTCTTGGCCTTGTAGGTGGTCTTGACCTCAGCGTCGTAGTGGCCG	
	CCGTCCTTCAGCTTCAGCCTCTGCTTGATCTCGCCCTTCAGGGCGCCGTCCTCG	
	GGGTACATCCGCTCGGAGGAGGCCTCCCAGCCCATGGTCTTCTTCTGCATTAC	
	GGGGCCGTCGGAGGGGAAGTTGGTGCCGCGCAGCTTCACCTTGTAGATGAAC	
	TCGCCGTCCTGCAGGGAGGAGTCCTGGGTCACGGTCACCACGCCGCCGTCCTC	
	GAAGTTCATCACGCGCTCCCACTTGAAGCCCTCGGGGAAGGACAGCTTC	
	AAGTAGTCGGGGATGTCGGCGGGGTGCTTCACGTAGGCCTTGGAGCCGT	
	ACATGAACTGAGGGGACAGGATGTCCCAGGCGAAGGGCAGGGGGCCA	
	CCCTTGGTCACCTTCAGCTTGGCGGTCTGGGTGCCCTCGTAGGGGCGGC	
	CCTCGCCCTCGCCCTCGATCTCGAACTCGTGGCCGTTCACGGAGCCCTCC	
	ATGTGCACCTTGAAGCGCATGAACTCCTTGATGATGGCCATGTTATCCTC	
	CTCGCCCTTGCTCACCATATGTGCCTCCTTAATTATAAAAGCCAGTCATTA	
	GGCCTATCTGACAATTCCTGAATAGAGTTCATAAACAATCCTGCATGATA	
	ACCATCACAAACAGAATGATGTACCTGTAAAGATAGCGGTAAATATATT	
	GAATTACCTTTATTAATGAATTTTCCTGCTGTAATAATGGGTAGAAGGTA	
	ATTACTATTATTATTGATATTTAAGTTAAACCCAGTAAATGAAGTCCATG	
	GAATAATAGAAAGAGAAAAAGCATTTTCAGGTATAGGTGTTTTGGGAA	
	ACAATTTCCCCGAACCATTATATTTCTCTACATCAGAAAGGTATAAATCA	
	ACAATTTCCCCGAACCATTATATTTCTCTACATCAGAAAGGTATAAATCA	
	TAAAACTCTTTGAAGTCATTCTTTACAGGAGTCCAAATACCAGAGAATGT	
	TTTAGATACACCATCAAAAATTGTATAAAGTGGCTCTAACTTATCCCAAT	
	AACCTAACTCTCCGTCGCTATTGTAACCAGTTCTAAAAGCTGTATTTGAGT	
	TTATCACCCTTGTCACTAAGAAAATAAATGCAGGGTAAAATTTATATCCT	
	TCTTGTTTTATGTTTCGGTATAAAACACTAATATCAATTTCTGTGGTTATA	
	CTAAAAGTCGTTTGTTGGTTCAAATAATGATTAAATATCTCTTTTCTCTTCC	
	AATTGTCTAAATCAATTTTATTAAAGTTCATATGTGCCTCCTTAAATAAAT	
	TCTGGAGTATGATAAATATATAAAAAATCAGAATGGTATGCTAAATGG	
	ATGATGTTTTGATTACAGTATGTAATTGATGGCATAAATGATGTTAACTT	
	ATGATTAACCCAGGTTTTATTACTTGTATGAACTATTACGAAATCAACTA	
	ATCCCCATAGTTTCTTAAAATCAAAAATAACGGTTTTATGTCCAAAAGTG	
	ATAAATTTAGTTTTGTGAATTATTTTATCTAAAGATAAAATGGCAATTATAAA	
	AGAATTTTTTTTATTGCGAATTGATTTAATTTTTTGCATCGCTATTTTTGAGGG	
	ATGAATAGCTCTTAAGCTCCTCATATATGCTAAATAATCTTCATCAAATTGTA	
	ATAAGTGTATTTTCTTTTTTAAATTAGCATCTACTCTGAGTTCTTTAATATCAT	
	GCTTACTTATCATATTGAAATCTGCTGTTTTAGGTAACTCTATTTTCTCAAAAT	
	AGATATTTAGTTCATATTTGTCGTTCATGCATGCCATG	

### Construction of the *pdpC*-null strain, complement and *emgfp* constructs

**Figure 1:**
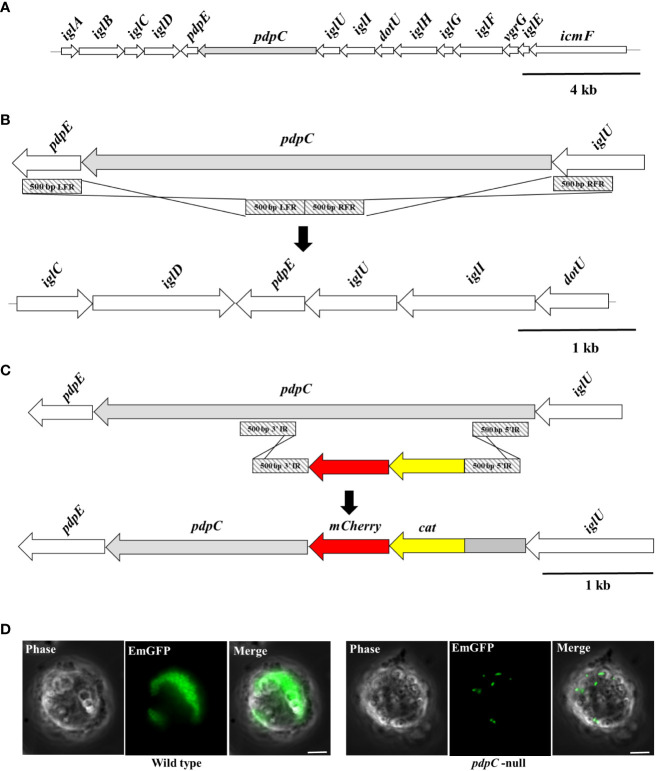
Construction of a *pdpC*-null strain of *F. tularensis* LVS. **(A)** The Francisella Pathogenicity Island (FPI). The FPI locus of *F. tularensis*, with *pdpC* shown in light grey. The FPI is duplicated in the *F. tularensis* chromosome. **(B)** Deletion of one copy of *pdpC* was achieved by homologous recombination of a deletion cassette (light grey diagonal stripes) created using 500 bp from either flank of *pdpC*. **(C)** The second chromosomal copy of *pdpC* was inactivated by insertion of a *cat mCherry* disruption cassette. **(D)** The *pdpC*-null strain is defective in intracellular replication. THP-1 human blood monocytes were incubated with fluorescently labelled wild type (at left) and *pdpC-*null (at right) *F. tularensis* LVS at an MOI of 100 for 2 hours. Cells were washed to remove extracellular bacteria and incubated for a further 24 hrs. Epi-fluorescence microscopy showed high levels of intracellular replication for the wild type but not for the *pdpC*-null strain.

The plasmids and oligonucleotides used in this study are listed in **Table 1**. Restriction endonucleases and Phusion polymerase were used according to the manufacturer’s instructions (New England Biolabs). There are two copies of *pdpC* in the *F. tularensis* LVS genome, FTL_0116 and FTL_1162. We used the unstable, integrating suicide vector, pJH1, and I-SceI endonuclease (Horzempa et al., 2010) to create an unmarked deletion of one copy of *pdpC*. The 500-bp flanking either side of *pdpC* were amplified by PCR using the primer pairs 0116F1/0116R1and 0116F2/0116R2, and then extension overlap PCR with the primer pair 0116F1/0116R2 was used to combine these two amplification products to generate a disruption cassette (**Figure 1**) that was cloned into pJHI to create pSC1. This plasmid was introduced into *F. tularensis* LVS by triparental mating (Horzempa et al., 2008a) and was excised by I-SceI restriction following the introduction of a plasmid, pGUTS (Horzempa et al., 2010) which encodes I-SceI and confers resistance to kanamycin. The pGUTS plasmid was cured by successive rounds of culture in non-selective CDM medium and kanamycin sensitive clones were identified by replica plating on chocolate II agar, with or without 10 µg ml^-1^ kanamycin. Primer pair 0116F1 and 0116R2 was used to screen for kanamycin sensitive clones that also contained the mutant allele of *pdpC*. During excision of the mutagenic plasmid, pSC1, a double stranded break is introduced by I-SceI and this can be resolved either by repair with a wild type copy of *pdpC* or with the mutant allele, therefore, it was anticipated that some clones would revert to wild type and that some would be merodiploid (containing one wild type and one mutant allele). A merodiploid clone was selected and the mutagenesis was repeated with the aim of deleting the second copy of *pdpC*. This repeated targeting was attempted several times, however, in each instance, only merodiploid clones were recovered. It is not clear why this was the case as a *pdpC*-null strain of *F. tularensis* LVS has been generated before using a similar deletion strategy (Lindgren et al., 2013). To inactivate the second copy of *pdpC* we switched to a different strategy, using a disruption cassette that could be selected based on antibiotic resistance. To do this a 2410 bp disruption cassette was designed with 500 bp of sequence flanking codons 183-723 of *pdpC* to allow for replacement of these codons with a chloramphenicol resistance gene (*cat*) and *mcherry* (which was included to allow fluorescence screening for the insertion but that was not used in this instance). The cassette was synthesized as a gBlock^®^ gene fragment (Integrated DNA Technologies) and amplified using primer pair SC25/26. This fragment was cloned into the *Xma*I *Sph*I site of pJH1 to create pSC7. This plasmid was introduced into *F. tularensis* LVS, exconjugants were selected based on resistance to kanamycin and chloramphenicol and PCR was used to confirm the insertion of pSC7 into the chromosome. Excision of pSC7 was achieved by introduction of pGUTS which was subsequently cured as described above and PCR using primer pair 0116F1/0116R2 was conducted to confirm insertion of the disruption cassette and that no wild-type allele of *pdpC* remained.

A complementation vector was constructed by amplifying a 4,048-bp fragment from *F. tularensis* LVS chromosomal DNA using the primer pair SC4/SC5 that contained the entire 3,987-bp coding sequence of FTL_0116/FTL_1162. This fragment also includes the short 39 bp intergenic region upstream of *pdpC* which is not expected to contain a promoter. The amplified fragment was cloned into the *Eco*RI-*Sal*I site of pABST (Robinson et al., 2015), a self-replicating vector containing the robust *Francisella groEp* promoter allowing for expression of *pdpC*, resulting in pCTK1.

To create a construct in which a *pdpC-emgfp* allele was expressed from the strong *Francisella FGRp* promoter (Horzempa et al., 2008b), *pdpC* was cloned into pSC18 (Cantlay et al., 2020), a vector containing *emgfp* and *FGRp*. The full-length *pdpC* gene, amplified from chromosomal DNA with SC4/SC5, was cloned into the *Eco*RI-*Sal*I site of pFNLTP8 (Maier et al., 2004) to generate pSC10. This vector was used as a template for the amplification of *pdpC* in two sections. Primer pair SC4/SC73 was used to amplify a 1559 bp fragment that includes codons 1-499 of *pdpC* and 41 bp upstream of the start codon of *pdpC* and introduces an *Eco*RI site. Primer pair SC71/72 was used to amplify a 2491 bp fragment that covers codons 499-1328 of *pdpC*. Primer, SC71, introduced an *Nde*I site and included a flexible LGPGE motif (Imai et al., 2000) that results in an LGPGEH linker between the last codon of *pdpC* and the start codon of *emgfp*. Codons 498-500 of *pdpC* are spanned by a naturally occurring *Sac*I site which was used to join the two fragments in a 3-molecule cloning into the *Eco*RI *Nde*I site of pSC18 to create pSC27. This construct was verified by sequencing (sequencing primers are listed in **Table 1**) to confirm that the *FGRp* and *pdpC* coding sequence was not altered and that *pdpC* and the flexible linker was in-frame with *emgfp*.

A vector expressing *ftsZ-emgfp* from the *FGRp* promoter was constructed in a similar way as described above, using primer pair SC31/33 to amplify a 1202 bp fragment including the coding sequence of Ftl_1907 (*ftsZ*) and a 43 bp upstream, intergenic region which was cloned into the *Eco*RI site of pSC18 to create pSC25.

The complementation vector (pCTK1), the *pdpC-emgfp* and *ftsZ-emgfp* vectors (pSC27 and pSC25), and the parent vectors used as controls were introduced into the wild-type and mutant strains by electroporation and selection on chocolate II agar supplemented with 10 µg ml^-1^ kanamycin.

### Fluorescence microscopy of Emgfp tagged *pdpC*-null *F. tularensis* LVS in THP-1 cells

The plasmid pKHEG (Cantlay et al., 2020) was mobilized into the *pdpC*-null mutant strain by electroporation. THP-1 human monocytes (ATCC) were cultured in RPMI 1640 medium (Gibco) supplemented with 10% FBS (Gemini Bioproducts), 25 mM HEPES (Corning), 2 mM Glutagro (Corning), 1mM Sodium Pyruvate (Gibco) and 0.05 mM 2-mercaptoethanol at 37°C with 5% CO_2_ to a density of 5x10^8^ cells ml^-1^, washed once in warm growth medium and incubated in 35-mm dishes at 37°C with 5% CO_2_ with wild type and mutant *F. tularensis* LVS strains containing *emgfp* on the plasmid pKHEG at an MOI of 100 for 24 hours. Samples were washed once with warm medium and fluorescently labelled *F. tularensis* LVS cells were visualized by epifluorescence microscopy. Uninfected THP-1 cells or THP-1 cells infected with wild-type or *pdpC*-null *F. tularensis* LVS containing an empty vector (pSC13) were used as negative controls for fluorescence.

### Gentamicin protection assays

Human erythrocytes were obtained from donated buffy coats (Vitalant) or whole blood (Lampire Biological Laboratories). Sheep erythrocytes were obtained from whole blood (Shiloh Farms Montadales). Erythrocytes were purified and assays were performed as described previously. Briefly, erythrocytes were purified from whole blood or donated buffy coats by adding 1 volume of PBS and were separated by density gradient centrifugation using Ficoll at 400x g for 30 minutes. Erythrocytes were washed once in PBS and then resuspended in McCoy’s 5A Medium supplemented with 25 mM HEPES, and 10% human serum (RBC Medium) prewarmed to 37°C (Horzempa et al., 2011). Erythrocytes were counted in a hemocytometer and adjusted to a concentration of 1 x10^7^ ml^-1^ in RBC medium. For the gentamicin protection assay, *F. tularensis* strains were grown overnight in CDM at 37°C, resuspended in prewarmed RBC medium and mixed with 1 x10^6^ erythrocytes to an MOI of between 12.5-50 as indicated in the text and figure legends. Erythrocytes and bacteria were incubated together at 37°C for 2 hours to allow invasion and then incubated for 1 hour with 50 µg ml^-1^ gentamicin (USB biologicals) under the same conditions. The gentamicin was removed by 2 washes with PBS and then erythrocytes were lysed in 0.02% SDS. Intracellular bacteria were enumerated by drip plating. The efficacy of the gentamicin treatment was tested in each experiment using controls in which bacteria, but no erythrocytes, were present. Each experiment was performed in triplicate.

### Fluorescence Microscopy

Imaging in all experiments was performed using an Olympus IX73 microscope equipped with a 100x NA. 1.30 Phase objective and an ORCA-Flash4.0 LT+ Digital CC11440-42U CMOS camera (Hamamatsu). Exposure times of 50 ms were used for each channel, and images were processed and analyzed using ImageJ (Schneider et al., 2012) and Fiji (Schindelin et al., 2012) All fluorescence images were adjusted for contrast and brightness using wild-type strains with or without an empty vector (pSC13 or pFNLTP8) as a reference to account for natural background fluorescence. For imaging of bacteria expressing *pdpC-emgfp* from the *FGRp* promoter, *F. tularensis* LVS strains were inoculated from plates in BHIc medium (Brodmann et al., 2017) or CDM at 37°C with agitation for 24 hours and spotted directly onto pads of 1% agarose in PBS. For imaging PdpC-Emgfp fluorescence in human erythrocytes, 3 x 10^6^ human erythrocytes were incubated in 35-mm dishes at 37°C with 5% CO_2_ with *F. tularensis* LVS containing *pdpC-emgfp* expressed from the *FGRp* promotor at an MOI of 100 for 3 hours. Samples were washed once with warm medium RBC medium and imaged in the 35-mm dishes after 24 hours. At least 4 fields of view were imaged in each experiment, and the experiments were repeated independently 4 times. At least 220 erythrocytes were scored in each experiment and a total of 2071 erythrocytes were scored over 4 experiments

### Assay for attachment of *F. tularensis* LVS to human erythrocytes

*F. tularensis* strains were incubated overnight in CDM at 37°C, resuspended in prewarmed RBC medium and mixed with 1 x10^6^ erythrocytes to an MOI of 100. Erythrocytes and bacteria were incubated together for 2 hours. Cells were pelleted at 100x g for 5 minutes and washed twice in PBS. Cells were then fixed in 2% paraformaldehyde and 0.1% glutaraldehyde in PBS for 1 hour at room temperature. Fixed cells were washed 3 times in PBS and then blocked in 2.5% BSA in PBS for 30 minutes at room temperature. Cells were probed with polyclonal mouse anti-*F. tularensis* Mab1 antibody (BEI Resources) at a concentration of 1:1000, overnight at 4°C. Cells were washed 3 times in PBS 0.05% Tween 20 and probed with Alexa Fluor 555 Donkey anti-Mouse IgG (Invitrogen) at a concentration of 1:1000 for 1 hour at room temperature. Cells were washed 3 times in PBS 0.05% Tween 20 and erythrocyte membranes were permeabilized with 0.1% Triton-X for 10 seconds, spun down for 5 minutes at 100x g and then washed 3 times in PBS. Cells were re-blocked in 2.5% BSA in PBS for 30 minutes at room temperature and then were re-probed with polyclonal mouse anti-*F. tularensis* Mab1 antibody (BEI Resources) at a concentration of 1:1000 for one hour at room temperature. Cells were washed 3 times in PBS 0.05% Tween 20 and probed with Alexa Fluor 488 Rat anti-Mouse IgG (Invitrogen) at a concentration of 1:1000 for 1 hour at room temperature. Cells were washed 3 times in PBS 0.05% Tween 20 and then washed once in PBS. Cells were spotted on pads of 1% agarose in PBS for imaging. Experiments were performed in triplicate at least 10 fields of view were analyzed for each condition. A minimum total of 325 erythrocytes were scored for each condition, with any bacteria that were dual labelled being scored as having attached, but not yet invaded.

### Assay for PdpC-EmGFP secretion in culture medium

*F. tularensis* LVS strains were scraped from chocolate II agar plates after 3 days of growth at 37°C with 5% CO_2_ and suspended in CDM and used to inoculate CDM broth cultures at a starting OD_600_ of 0.1. After 24 hours growth at 37°C with 5% CO_2_ bacteria were pelleted by centrifugation and supernatants were collected and filter sterilized using a 0.22 µm syringe filter (Millipore) to remove any remaining bacteria. Fluorescence of 6 replicates was measured in an Eppendorf AF2200 plate reader with excitation/emission set to 485/535 nm with a 10 nm bandwidth for both.

### Assay for PdpC-EmGFP secretion in erythrocytes

To analyze PdpC-EmGFP fluorescence in human erythrocyte lysates, 1 x10^7^erythrocytes were mixed with *F. tularensis* LVS strains that had been grown to stationary phase overnight in CDM at an MOI of 100. Erythrocytes and bacteria were incubated for 24 h at 37°C with 5% CO_2_. Cells were spun down at 300 x g for 5 min and the pellets were resuspended in 1 ml of 0.02% SDS in PBS to lyse the erythrocytes. The lysates and supernatant were filtered through a 0.22 µm syringe filter (Millipore) to remove bacteria. The fluorescence of 200 µl volumes of lysate and supernatant was measured in an Eppendorf AF2200 plate reader with excitation/emission set to 485/535 nm with a 10 nm bandwidth for both. The fluorescence from either 0.02% SDS in PBS (for lysates) or Red Blood Cell Medium (for supernatants) alone was measured and this was subtracted to give background adjusted values. The experiment was repeated independently with four biological replicates, and erythrocytes incubated without *F. tularensis* LVS strains were used as a negative control.

### Statistical analyses

Data were analyzed using GraphPad Prism software. The tests used and the *P* values obtained are presented in the text and figure legends.

## Results

### Construction of *pdpC-*null mutant

The T6SS has previously been shown to be required for erythrocyte invasion by *F. tularensis* LVS (Schmitt et al., 2017). We wanted to test if PdpC, a secreted effector of the T6SS (Eshraghi et al., 2016) was required for erythrocyte invasion by generating a strain of *F. tularensis* LVS that was a mutant for *pdpC*. There are two identical copies of *pdpC* in the *F. tularensis* LVS genome, encoded in the duplicated Francisella Pathogenicity Island (**Figure 1A**). In order to create a *pdpC-*null strain, one copy of *pdpC* was deleted using homologous recombination of flanking sequences cloned into an excisable, unstable plasmid as previously described (Horzempa et al., 2010) (**Figure 1B**). The second copy of *pdpC* was then disrupted using a similar strategy that replaced codons 183-723 of *pdpC* with a *cat mcherry* disruption cassette (**Figure 1C**). Insertion of the disruption cassette created a frameshift to prevent read through from the *cat mcherry* cassette that could result in translation of any downstream PdpC sequence. The first 182 codons of *pdpC* were left intact to minimize any potential polar effects, however the insertion of the disruption cassette introduced a stop codon that would allow only the production of the C- terminal of PdpC with no read through into the *cat mcherr*y genes. PCR was used to confirm that no wild-type copies of *pdpC* remained and Western blotting confirmed that only a ~21.8 kDa truncated C-terminal portion of PdpC was detected in the *pdpC*-null strain (data not shown). To ensure that the activity of PdpC had been fully abrogated we introduced a fluorescent reporter into the *pdpC*-null strain and assayed its ability to proliferate within host cells. Wild type and *pdpC*-null bacteria expressing *emgfp* were incubated with THP-1 leukemic monocytes (Tsuchiya et al., 1980) at an MOI of 100. After 2 hours of incubation, the THP-1 cells were washed to remove extracellular bacteria and infection and intracellular proliferation was monitored using epi-fluorescence microscopy. After 24 hours, intracellular proliferation of *F. tularensis* LVS was observed in almost all the THP-1 cells incubated with wild type bacteria and many cells had died. For THP-1 cells incubated with the *pdpC*-null strain there was no obvious cell death and, whilst fluorescent bacteria could be observed in cells (indicating that uptake was not affected in this strain), there was no evidence of intracellular proliferation (**Figure 1D**). We conclude, therefore, that the *pdpC-*null strain is not competent for intracellular proliferation, indicating that the function of PdpC has been abolished in this strain.

We have previously shown that OpiA, another effector protein of the T6SS, whilst not required for erythrocyte invasion, does affect growth and morphology of *F. tularensis* LVS (Cantlay et al., 2020). However, we saw no defect in either growth or morphology for the *pdpC*-null strain compared to the wild type when grown in standard broth culture in Chamberlain’s Defined Medium (data not shown) and therefore conclude that *pdpC* does not influence bacterial physiology under the conditions tested.

### PdpC is required for erythrocyte invasion

**Figure 2:**
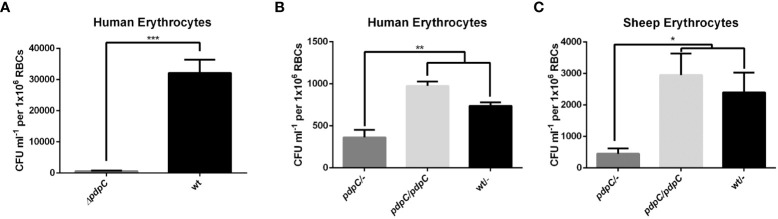
PdpC is required for erythrocyte invasion. **(A)** Human erythrocytes were incubated with either wild type or *pdpC*-null *F. tularensis* LVS at an MOI of 50 for 2 hours, cells were then treated for 1 hour with gentamicin to kill extracellular bacteria. Erythrocytes were washed, lysed and intracellular bacteria were enumerated by drip plating. The CFU ml^-1^ per 1x10^6^ Red Blood Cells (RBCs) was significantly lower for the *pdpC*-null strain (***p > 0.0001 determined by unpaired t-test with Welch’s correction). **(B)** Introduction of a copy of *pdpC*, *in trans*, restores invasion in human erythrocytes. Erythrocytes were incubated with the indicated strains at an MOI of 12.5 and invasion was tested by gentamicin protection assay as described above. The CFU ml^-1^ per 1x10^6^ Red Blood Cells (RBCs) was significantly lower for the *pdpC*-null strain than either the wild type or the *pdpC-*null strain containing a complementation vector (**p > 0.01 determined by Ordinary 1 way ANOVA with Holm-Sidak's multiple comparisons test). **(C)** PdpC is required for invasion of Sheep erythrocytes. Sheep erythrocytes were incubated with the indicated strains at an MOI of 100 and invasion was tested by gentamicin protection assay as described above. The CFU ml^-1^ per 1x10^6^ Red Blood Cells (RBCs) was significantly lower for the *pdpC*-null strain than either the wild type or the *pdpC-*null strain containing a complementation vector (*p > 0.05 determined by Ordinary 1 way ANOVA with Holm-Sidak's multiple comparisons test). The averages of at least 3 biological replicates, ± the standard error, are shown and for each biological replicate bacteria incubated without erythrocytes were used as a negative control and this background was subtracted from the means.

To test whether *pdpC* is required for erythrocyte invasion we employed a gentamicin protection assay as previously described (Horzempa et al., 2011; Schmitt et al., 2017). Wild type and *pdpC*-null *F. tularensis* LVS strains were incubated with human erythrocytes for 2 hours at an MOI of 50. Erythrocytes were then incubated for 1 hour in PBS with 50 µg ml^-1^ gentamicin to kill any extracellular bacteria. The erythrocytes were washed twice in PBS and then lysed, and the number of intracellular bacteria were enumerated by plating serial dilutions to count colony forming units (CFUs). This experiment was conducted 3 times with 3 biological replicates and the *pdpC*-null strain was strongly affected in its ability to invade human erythrocytes (*p* > 0.0001 determined by unpaired t-test with Welch’s correction) (**Figure 2A**). To verify that this defect in erythrocyte invasion was indeed caused by the mutation of *pdpC* and not any polar effects or unlinked mutations we conducted complementation studies. A copy of *pdpC* expressed, *in trans*, from the *Francisella FGRp* promoter (Horzempa et al., 2008b) was introduced into the mutant and wild-type strains and erythrocyte invasion assays were performed as described above, this time with an MOI of 12.5. In these experiments the *pdpC*-null strain containing an empty vector showed significantly less invasion of human erythrocytes than either the wild-type containing the empty vector or the *pdpC*-null strain containing the complementation vector (*p*> 0.01 determined by ordinary 1 way ANOVA with Holm-Sidak’s multiple comparisons test) (**Figure 2B**). The same experiments were performed again using erythrocytes from sheep and similar results were observed (*p*> 0.05 determined by ordinary 1 way ANOVA with Holm-Sidak’s multiple comparisons test) (**Figure 2C**). These results demonstrate that *pdpC* is required for invasion of both human and sheep erythrocytes under the conditions tested.

### PdpC is involved in attachment of *F. tularensis* LVS to erythrocytes during invasion

**Figure 3:**
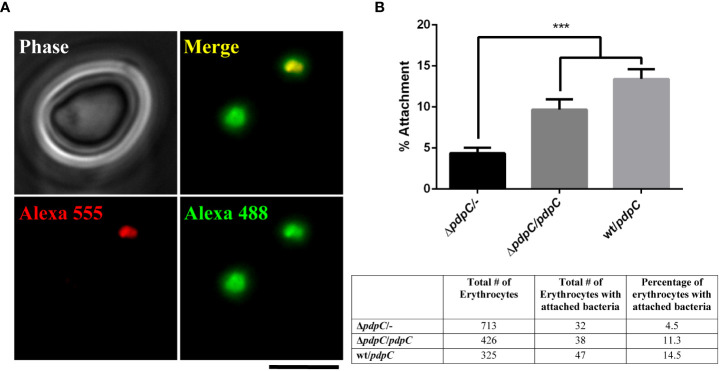
PdpC is required for efficient attachment to erythrocytes. *F. tularensis* strains were incubated with 1x 10^6^ human erythrocytes at an MOI ~100 for 2 hours. Cells were pelleted, washed and DIF microscopy was used determine the number erythrocytes with attached bacteria. **(A)** An example of DIF microscopy. A red blood cell incubated with wt *F. tularensis* LVS containing a complementation plasmid. Bacterial cells attached to the surface are labeled with both red and green secondary antibodies. A bacterial cell that has invaded the red blood cell is labelled only in green. The scale bar represents 5 µm. **(B)** Dual labeled erythrocytes were scored for the presence of at least one attached *F. tularensis* LVS cell. The mean percentage of erythrocytes with attached bacteria is shown. Significantly fewer erythrocytes with attached bacteria were seen in the samples incubated with the *pdpC*-null mutant containing an empty vector than either the *pdpC*-null mutant containing a complementation vector with a copy of *pdpC* under the control of the strong *groEp* promoter, or the wild type containing the same complementation vector (***p > 0.001 determined by 1-way ordinary ANOVA with Tukey’s multiple comparisons test). The averages of 3 biological replicates, ± the standard error, are shown. For each experimental condition at least 325 erythrocytes were scored in total for attachment of bacteria and the total number of erythrocytes scored is shown in the table.

Having determined that *pdpC* is required for the invasion of erythrocytes, we next used double immuno-fluorescence microscopy (DIF Microscopy) (Clemens et al., 2004; Horzempa et al., 2011) to further investigate the interaction between human erythrocytes and the *pdpC*-null strain. This technique involves incubating erythrocytes and bacteria, fixing the cells and then sequentially probing with fluorescently labelled antibodies before and after permeabilization of the erythrocyte membrane. Bacteria that are on the surface of the erythrocytes at the time of fixation will be labelled with both fluorescent antibodies, whilst any bacteria that had successfully invaded an erythrocyte will only be labelled after permeabilization of the erythrocyte membrane. This allows us to distinguish between attached bacteria and those ones that had invaded (**Figure 3A**). Initial experiments, performed in triplicate, comparing wild type *F. tularensis* LVS to the *pdpC-*null strain showed that, consistent with the gentamicin protection assays, fewer invading bacteria were detected for the *pdpC-*null strain but also that fewer *pdpC*-null bacteria were attached to the red blood cells (data not shown). To ensure that the observed results were due to the absence of PdpC and not any unlinked or polar effects, complementation studies were conducted. For these experiments, *F. tularensis* strains, grown overnight at 37°C in CDM were resuspended in RBC medium and incubated with 1x 10^6^ human erythrocytes at an MOI ~100 for 2 hours. Cells were pelleted, washed and DIF microscopy was used determine the number of erythrocytes with attached bacteria. Significantly fewer erythrocytes with attached bacteria were seen for samples incubated with the *pdpC*-null mutant containing an empty vector than either the *pdpC*-null mutant containing a complementation vector with a copy of *pdpC* under the control of the strong *groEp* promoter, or the wild type containing the same complementation vector (*p* > 0.01 determined by 1-way ordinary ANOVA with Tukey’s multiple comparisons test) (**Figure 3B**). Based on these results, we conclude that PdpC is required for efficient attachment of *F. tularensis* LVS to human erythrocytes.

### PdpC-EmGFP localizes as discrete, punctate foci in *F. tularensis* LVS and is secreted into culture medium

**Figure 4:**
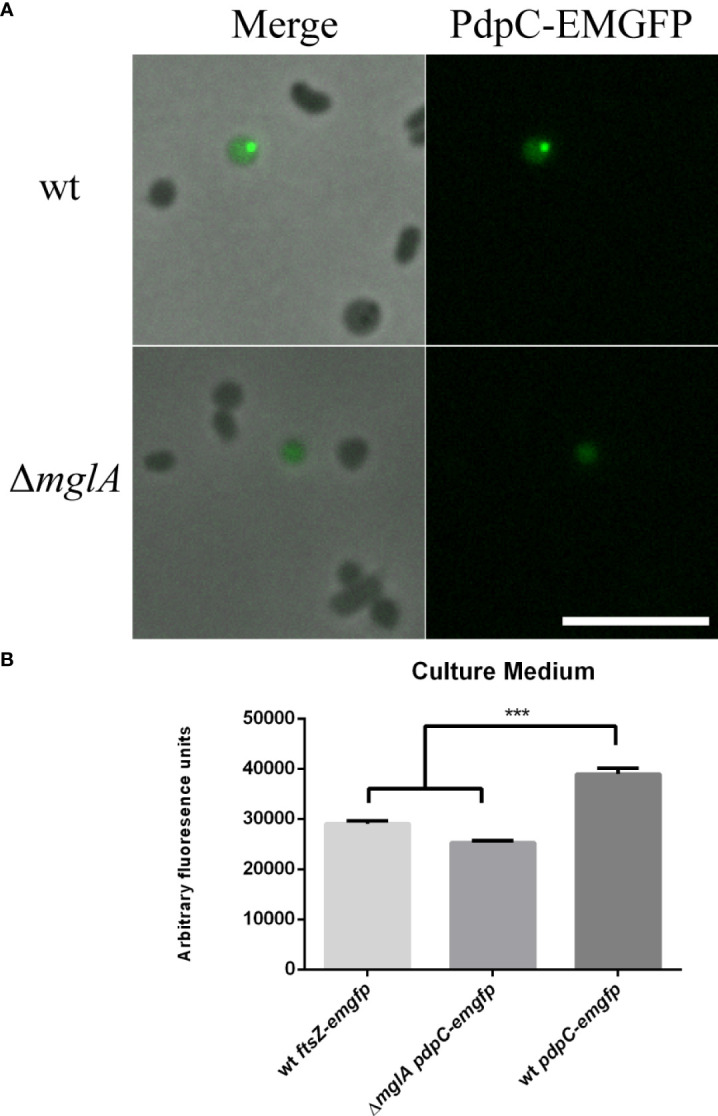
PdpC-EMGFP localizes as foci in *F. tularensis* and localization and secretion is dependent on *mglA*. *F. tularensis* LVS strains containing a copy of *pdpC-emgfp*, *in trans*, under the control of the *FGRp* promoter were grown for 24 hours in CDM at 37°C. **(A)** Punctate foci could be seen in a small subset of wt cells (top) whereas only diffuse fluorescence was seen for an *mglA* mutant (bottom). Scale bar equals 5 µm. **(B)** Filter sterilized supernatant from the wt containing *pdpC-emgfp* showed significantly higher fluorescence than supernatants from either an *mglA-* null mutant or the wt strain expressing *ftsZ*-*emgfp* , a cytosolic protein which is not secreted (***p < 0.001 determined by ordinary 1-way ANOVA with Tukey’s multiple comparisons test). The averages of six independent experiments are shown ± the standard error.

Next, we generated a strain of *F. tularensis* LVS expressing a *pdpC-emgfp* allele. The coding sequence of *pdpC* and the upstream, intergenic region was cloned in-frame with *emgfp* and downstream of the strong *Francisella FGRp* promoter in a vector that was mobilized into wild type *F. tularensis LVS*. This created a strain that expresses both wild type *pdpC* under native conditions, and the *pdpC*-*emgfp* allele under the control of the *FGRp* promoter. *F. tularensis* LVS cells expressing the recombinant *pdpC*-*emgfp* allele were grown for 24 hours in CDM at 37°C and cells were spotted directly onto pads of 1% agarose in PBS for visualization by epi-fluorescence microscopy. Three independent experiments were conducted, and for each experiment a small subset (<0.1%) of cells had a single punctate fluorescence focus (**Figure 4A**). Next, we introduced the *pdpC-emgfp* allele into *F. tularensis* LVS Δ*mglA*, a strain that contains a null mutation of *mglA* which encodes a transcription factor required for expression of the T6SS genes of the FPI (Schmitt et al., 2017). In this strain, we observed only a diffuse fluorescence in a similar small subset of cells (**Figure 4A**). To test if PdpC-EMGFP was being secreted into the culture medium we collected supernatants from wild type and Δ*mglA F. tularensis* LVS strains, filter sterilized this material to remove any bacteria, and measured fluorescence using a plate reader. We also included the wild type LVS strain expressing *ftsZ-emgfp* from the FGRp promoter. FtsZ is a cytosolic protein involved in cell division (Bi and Lutkenhaus, 1991; Coltharp and Xiao, 2017) and is not a substrate of the T6SS or any other secretion system. There was significantly higher fluorescence in supernatants for wild type *F. tularensis* LVS expressing *pdpC-emgfp* than for either the Δ*mglA* or the wild type strain expressing *ftsZ-emgfp* (*p* > 0.001 determined by 1-way ordinary ANOVA with Tukey’s multiple comparisons test), suggesting that PdpC-EMGFP is being secreted into the culture medium in an *mglA*-dependent fashion (**Figure 4B**).

### PdpC-EmGFP accumulates in erythrocytes during invasion assays

**Figure 5:**
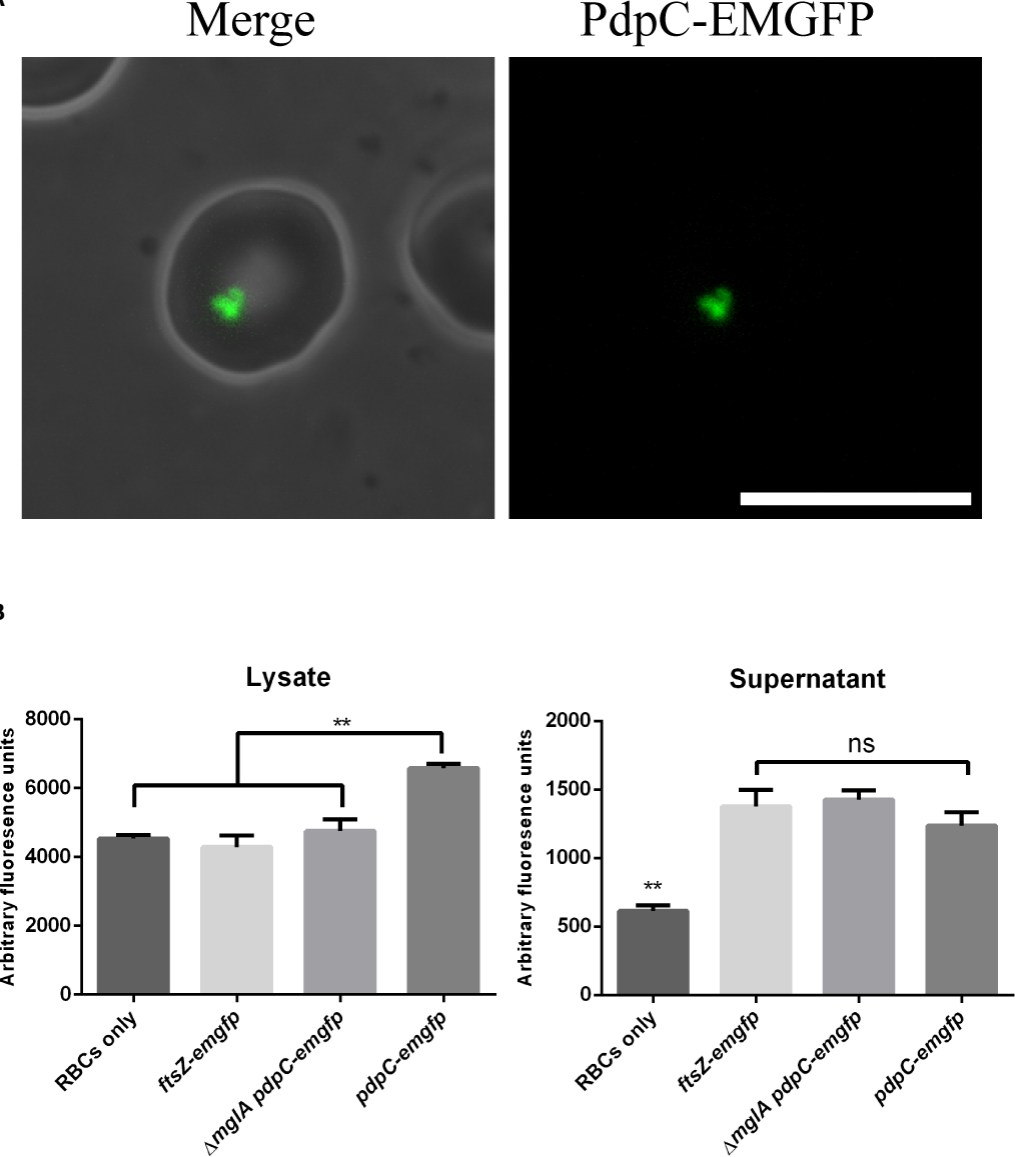
PdpC-EMGFP is secreted during erythrocyte invasion. **(A)** *F. tularensis* LVS strains expressing *pdpC-emgfp* were incubated with 3 x10^6^ erythrocytes at an MOI of 100 for 24 hours and analysed by epi-flouresnce microscopy.PdpC-EmGFP fluorescence could be seen in approximately 1% of erythrocytes. A total of 2071 erythrocytes were scored for fluorescence over 4 independently conducted experiments. Scale bar equals 10 µm. **(B)** *F. tularensis* LVS strains containing either a vector with *pdpC-emgfp* or a vector expressing *ftsZ-emgfp* from *FGRp* were incubated with 1 x10^7^ erythrocytes at an MOI of 100 for 24 hours. Erythrocytes were pelleted and lysed, filter sterilized to remove any bacteria and the fluorescence of the lysate (top panel) and supernatant (lower panel) was measured. The fluorescence in lysates from erythrocytes incubated with wt *F. tularensis* LVS expressing *pdpC*-*emgfp* was significantly higher than that from erythrocytes incubated with either an *mglA* mutant expressing *pdpC-emgfp* or the wt strain expressing *ftsZ-emgfp* (**p < 0.05 determined by ordinary 1-way ANOVA with Tukey’s multiple comparisons test), whilst there was no difference (ns) in the fluorescence of supernatant from strains expressing either *pdpC-emgfp or ftsZ-emgfp*. The averages of four independent experiments are shown ± the standard error.

We were surprised at how few fluorescence foci were observed in broth cultures of *F. tularensis* LVS expressing *pdpC*-*emgfp* but reasoned that, under the conditions tested, there may not have been high expression of the pathogenicity island genes. Because we know that the T6SS is involved in erythrocyte invasion and also that PdpC is required for both efficient attachment and invasion we sought to visualize PdpC-EmGFP during an erythrocyte invasion assay. *F. tularensis* LVS strains were incubated overnight in CDM and then resuspended in RBC medium and incubated with 3 x 10^6^ human erythrocytes for 3 hours in 35 mm dishes. Erythrocytes were washed once in RBC medium to remove any extracellular bacteria. We did not see any significant increase in PdpC-EmGFP fluorescence directly after the 3-hour incubation period, however, some fluorescent foci were visible at the periphery of erythrocytes (data not shown) presumably associated with bacterial cells attached to or invading erythrocytes. For erythrocytes that were incubated a further 24 hours, fluorescence could be observed inside 1.18% (± 0.16%) of cells (**Figure 5A**). This fluorescence did not appear to be discrete foci associated with invaded bacteria, suggesting that PdpC-EmGFP fluorescence accumulates in red blood cells during invasion. To test this further we measured the fluorescence of erythrocyte lysates 24 hours after incubation with *F. tularensis* LVS expressing *pdpC*-*emgfp*. Erythrocyte invasion assays were conducted as described above and after 24 hours red blood cells were lysed, filtered to remove any intracellular bacteria and fluorescence was measured by plate reader. The fluorescence in lysates from red blood cells incubated with wild type *F. tularensis* LVS expressing *pdpC*-*emgfp* was significantly higher than that from erythrocytes incubated with either the Δ*mglA* mutant expressing *pdpC-emgfp* or the wild type strain expressing *ftsZ-emgfp* (*p* > 0.01 determined by 1-way ordinary ANOVA with Tukey’s multiple comparisons test). There was no significant difference in the fluorescence of supernatant from any of the strains tested (**Figure 5B**). These results indicate the PdpC-EmGFP is specifically being secreted into red blood cells during infection and is consistent with our microscopic observations.

## Discussion

In addition to species of protozoans that parasitize blood (Flaherty et al., 2018) there are also several bacteria that have been shown to be able to invade erythrocytes. *Bartonella* spp. are gram-negative, host-restricted mammalian pathogens that can infect both endothelial cells and erythrocytes (Schülein et al., 2001). *Anaplasma marginale*, also a gram-negative bacterium, causes bovine anaplasmosis (Kocan et al., 2007). Additionally, the *Mycoplasma* species, *M. suis* and *M*. *gallisepticum* have been shown to invade host erythrocytes (Vogl et al., 2008; Groebel et al., 2009). In each case, the ability to invade host red blood cells represents a specific evolutionary adaptation that contributes to pathogenicity and persistence. Elucidating the mechanisms through which *F. tularensis* is able to invade host erythrocytes, therefore, will be important in understanding the pathogenicity and ecology of this tier 1 select agent.

Given that the T6SS is required for erythrocyte invasion (Horzempa et al., 2011; Schmitt et al., 2017) we are interested in the role played by the secreted effector proteins. In this study, we identify PdpC as the first known effector protein of the T6SS to be required for erythrocyte invasion in *F. tularensis* LVS. Using a gentamicin protection assay we report here that PdpC is required for efficient invasion of both human and sheep erythrocytes *in vitro*. We conducted 3 different sets of experiments, the first used blood isolated from buffy coats comparing the wild type with the *pdpC*-null strain (Fig 2A). In subsequent experiments we used whole blood sourced either from human or sheep (Fig 2B,C). Interestingly, we saw much higher levels of invasion in the first set of experiments (**Figure 2A**). We speculate that the most likely reason for this difference is due to the difference in source of blood for the experiments. The whole blood extracts obtained were at least stored for one day more prior to use than the erythrocytes isolated from buffy coats and the membrane of erythrocytes can be altered over time (Smith, 1987; Nikolovski et al., 2012) and our unpublished observations suggest that erythrocyte storage negatively influences invasion. However, we cannot rule out the possibility that the presence of complementation or control vectors in strains contributed to the observed differences. Additionally, despite our efforts to standardize the MOI by normalizing an inoculum from stationary phase cultures by optical density, we saw that we obtained different MOIs in each set of experiments. We are not sure why this was the case although we speculate that there can be differences in the culturability and viability of *F. tularensis* LVS cultures due to changes in physiology after cells reach stationary phase in broth culture or on agar plates used to inoculate broth cultures. Future experiments to examine the influence of growth conditions, bacterial viability and culturability and medium dependent effects on erythrocyte invasion will be required to address this. Even though we observed some variability between these three sets of experiments they all support the conclusion that *pdpC* is required for invasion of red blood cells under the conditions tested.

Of all the secreted effector proteins of the T6SS in *Francisella* spp. so far characterized, PdpC has been shown to play a major role in pathogenesis, specifically being required for efficient escape from the phagosome during host cell infection. In the highly virulent *F. tularensis* SCHU S4 strain, a mutant lacking both copies of *pdpC* was delayed in escape from mouse macrophages *in vitro* and *in vivo*, following intranasal challenge, was able to disseminate to spleen and liver in mice but ultimately was not able to cause severe disease (Long et al., 2013). In *F. novicida*, *pdpC* had the strongest effect on virulence (Eshraghi et al., 2016; Brodmann et al., 2017) whilst single mutants for *pdpD*, *opiA* and *opiB* did not have a strong effect by themselves. However, mutants lacking *pdpC*, *pdpD*, *opiA* and *opiB* in combination were significantly reduced in virulence (Eshraghi et al., 2016). Both *pdpC* and *pdpD* are required for full activation of the AIM2 inflammasome indicative of phagosomal escape and intracellular replication (Brodmann et al., 2017). During macrophage infection, then, the secreted effector proteins of the T6SS in *Francisella* spp. appear to exert cumulative effects and are likely to work in combination for full virulence. This does not appear to the be case for erythrocyte invasion, as we have recently characterized the function of OpiA in *F. tularensis* LVS and it did not have any effect on erythrocyte invasion (Cantlay et al., 2020). OpiA was of interest because it has been identified as a PI (3) kinase and had been shown to act on host macrophage membranes (Ledvina et al., 2018). Because red blood cells do not have any endocytic capability, we predicted that an interaction with molecules of the erythrocyte membrane will be important for invasion. We previously reasoned that OpiA may be able to act directly on the erythrocyte membrane to facilitate invasion (Cantlay et al., 2020), however this protein was not required. During infection of macrophages OpiA functions to delay phagosome maturation, thereby facilitating escape of the bacteria, but only in a Δ*pdpC* mutant background (Ledvina et al., 2018) suggesting that PdpC and OpiA may function in a common pathway (Ledvina et al., 2018), but that remains to be determined regarding erythrocyte invasion.

One possibility is that PdpC can interact directly with the erythrocyte membrane. Other parasites of red blood cells, such as *P. falciparum*, *Bartonella* spp. and *Mycoplasma* spp. have all been observed to deform the red blood cell membrane during invasion (Hendrix and Kiss, 2003; Vogl et al., 2008; Sisquella et al., 2017). However, given the data suggesting that PdpC is secreted into the erythrocyte, and is required for attachment, it is possible that this bacterial protein facilitates a stronger interaction between the host and pathogen cells. Two potential interactors could be the erythrocyte membrane-spanning protein, Band 3, and the major cytoskeletal protein, spectrin. Consistent with this potentiality, Band 3 and spectrin have previously been implicated in erythrocyte invasion by *F. tularensis* (Schmitt et al., 2017). Moreover, other pathogens that invade red blood cells (*P. falciparum* and *Bartonella* sp.) likely utilize Band 3 as a receptor protein (Deng et al., 2012; Almukadi et al., 2019). Another possibility could be the complement receptor since intact serum is required for erythrocyte invasion. Future work will focus on identifying host erythrocyte interacting partners of PdpC.

Whether it is through direct contact with the membrane or *via* protein-protein interactions, PdpC is required at an early stage of erythrocyte invasion with attachment being significantly reduced in the *pdpC*-null strain. This is consistent with previous research that showed that the T6SS was required for erythrocyte invasion in *F. tularensis* LVS (Schmitt et al., 2017). Moreover, it indicates that the structural components of the T6SS alone are not sufficient for attachment, in contrast to *Bartonella* spp. which utilize a Type Four Secretion system (T4SS) for attachment to host erythrocytes in a highly host-specific manner (61, 68). Because PdpC is required for invasion in both human and sheep erythrocytes and is required for phagosomal escape during infection of macrophages suggests that the target or mode of action for PdpC is present in these different hosts and cell types. However, PdpC is a large protein, ~ 156 kDa (Nano and Schmerk, 2007), and the possibility remains that this polypeptide facilitates multiple functions.

Finally, we used a fluorescence fusion of PdpC to EmGFP to localize PdpC in *F. tularensis* LVS and in human erythrocytes. We saw that PdpC-EmGFP localized as discrete puncta in a very small subset of cells. The localization was reminiscent to that reported for the T6SS sheath protein, IglA, in *F. novicida* (Clemens et al., 2015; Brodmann et al., 2017) and the secreted effector OpiA from *F. tularensis* LVS (Cantlay et al., 2020). Expression of the FPI genes, including *pdpC*, is driven from a promoter regulated by MglA/SspA and PigR in response to stress, such as that encountered inside a host cell (Wrench et al., 2013; Cuthbert et al., 2015; Cuthbert et al., 2017; Travis et al., 2021). Due to this complex regulation we chose to express *pdpC-emgfp* from a strong *Francisella* promoter, *FGRp*. Given that the observed PdpC-EmGFP localization in broth culture is dependent on *mglA*, it is possible that the localization of PdpC-EMGFP is dependent on structural components of the T6SS or even other effector proteins, including native PdpC, that would not be present in the *mglA* mutant. During erythrocyte invasion however, when expression of the FPI is presumably high, we saw an accumulation of PdpC-EmGFP fluorescence inside erythrocytes. No increase in fluorescence was observed in the medium for wild type or Δ*mglA* strains expressing *pdpC-emgfp* or a wild type strain expressing *ftsZ-emgfp* from the same promoter. Because erythrocytes have no capacity for pinocytosis (Blanton et al., 1968; Maurin and Raoult, 2001) this indicates that PdpC-EmGFP is specifically being secreted into the erythrocytes. Due to the requirement of PdpC for attachment to erythrocytes, we expected to see PdpC-EmGFP fluorescence at an early time point during invasion. However, this was not the case and instead we saw PdpC-EmGFP associated fluorescence only after 24 hrs. In *Bartonella* sp. bacteria invading erythrocytes have been observed to reside in a vacuolar membrane (Schülein et al., 2001), if *F. tularensis* does the same, secretion and accumulation of PdpC-EmGFP into this space may explain the observed pattern of fluorescence localization. It is known that oxidizing environments, such as the periplasmic space or the red blood cell itself, can affect the maturation of green fluorescent protein (Feilmeier et al., 2000) and so PdpC-EmGFP may be present at earlier time points but can only be observed in erythrocytes under conditions favorable to maturation and correct folding of the fluorescent tag. We are also not able to exclude the possibility that the micrographs showing increased PdpC-EMGFP fluorescence inside erythrocytes also includes PdpC-EMGFP fluorescence inside bacterial cells. However, by filtering bacteria from the erythrocyte lysates we present clear evidence that PdpC-EMGFP is secreted into red blood cells during invasion. Future experiments using confocal fluorescence microscopy will be required to study the fate of PdpC during erythrocyte invasion in greater resolution.

The fluorescence localization experiments showed an accumulation of PdpC-EmGFP fluorescence in approximately 1% of erythrocytes. This is consistent with the data from gentamicin protection assays and DIF microscopy from this and previous studies (Horzempa et al., 2011; Schmitt et al., 2017) which suggest that in any given population of erythrocytes 1-5% are successfully invaded. For *Bartonella* sp. an invasion rate of 0.017-0.059% has been reported (Schülein et al., 2001) and for *M. gallisepticum* a similar rate of 0.05% has been observed (Vogl et al., 2008). We have consistently observed rates of invasion of between 1-5% suggesting that *F. tularensis* LVS is relatively well adapted to erythrocyte invasion compared to other bacterial species.

Identifying the requirement of PdpC for erythrocyte invasion is an important step in understanding how *F. tularensis* gains entry into red blood cells. However, the function of PdpC in this process remains a mystery. Future work to identify and characterize protein or membrane binding interactions of PdpC will be critical to understanding the exact mechanism of action. The mutant and recombinant strains generated for this study will be useful tools to help dissect the role of PdpC in host cell interactions and in future studies to determine the effects of other secreted protein effectors of the T6SS on erythrocyte invasion.

## Data availability statement

The original contributions presented in the study are included in the article/supplementary material. Further inquiries can be directed to the corresponding author.

## Author contributions

SC and JH conceived and designed the experiments. SC and CK performed the experiments. SC and JH analyzed the data. SC and JH wrote the manuscript. All authors contributed to the article and approved the submitted version

## Funding

This study was funded by the National Institutes of Health, Heart Lung and Blood Institute (1R15HL147135) and an Institutional Development Award (IDeA) from the National Institute of General Medical Sciences of the National Institutes of Health (P20GM103434), which funds WV-INBRE program. This research was made possible by NASA West Virginia Space Grant Consortium training grant NNX15A101H.

## Conflict of interest

The authors declare that the research was conducted in the absence of any commercial or financial relationships that could be construed as a potential conflict of interest.

## Publisher’s note

All claims expressed in this article are solely those of the authors and do not necessarily represent those of their affiliated organizations, or those of the publisher, the editors and the reviewers. Any product that may be evaluated in this article, or claim that may be made by its manufacturer, is not guaranteed or endorsed by the publisher.
